# DAFT-Net: Dual Attention and Fast Tongue Contour Extraction Using Enhanced U-Net Architecture

**DOI:** 10.3390/e26060482

**Published:** 2024-05-31

**Authors:** Xinqiang Wang, Wenhuan Lu, Hengxin Liu, Wei Zhang, Qiang Li

**Affiliations:** 1 Tianjin Key Lab of Cognitive Computing and Application, College of Intelligence and Computing, Tianjin University, Tianjin 300350, China; 13502076951@163.com; 2 School of Software and Communication, Tianjin Sino-German University of Applied Sciences, Tianjin 300350, China; 3 College of Intelligence and Computing, Tianjin University, Tianjin 300072, China; 4 School of Microelectronics, Tianjin University, Tianjin 300072, China; 5 Nanjing Research Institute of Electronic Engineering, Nanjing 210023, China

**Keywords:** tongue contour, feature extraction, image segmentation, entropy optimization, real-time, dual attention mechanism

## Abstract

In most silent speech research, continuously observing tongue movements is crucial, thus requiring the use of ultrasound to extract tongue contours. Precisely and in real-time extracting ultrasonic tongue contours presents a major challenge. To tackle this challenge, the novel end-to-end lightweight network DAFT-Net is introduced for ultrasonic tongue contour extraction. Integrating the Convolutional Block Attention Module (CBAM) and Attention Gate (AG) module with entropy-based optimization strategies, DAFT-Net establishes a comprehensive attention mechanism with dual functionality. This innovative approach enhances feature representation by replacing traditional skip connection architecture, thus leveraging entropy and information-theoretic measures to ensure efficient and precise feature selection. Additionally, the U-Net’s encoder and decoder layers have been streamlined to reduce computational demands. This process is further supported by information theory, thus guiding the reduction without compromising the network’s ability to capture and utilize critical information. Ablation studies confirm the efficacy of the integrated attention module and its components. The comparative analysis of the NS, TGU, and TIMIT datasets shows that DAFT-Net efficiently extracts relevant features, and it significantly reduces extraction time. These findings demonstrate the practical advantages of applying entropy and information theory principles. This approach improves the performance of medical image segmentation networks, thus paving the way for real-world applications.

## 1. Introduction

Ultrasound technology, characterized by its cleanliness, safety, and cost-effectiveness, facilitates the imaging of the tongue and mouth. The precise extraction of tongue contours from these images assists physicians in monitoring the vocalization of patients with speech impairments due to diseases or speech disorders. It also serves as a reference for language pronunciation in sensitive contexts [1] and allows for the integration of tongue features as biological signals into silent speech interfaces [2]. Silent speech technology offers a means of communication for those who have lost their voice due to illness or accident. Additionally, it provides clear speech input in high-noise environments. In silent speech analysis, the accuracy of ultrasound tongue contour extraction directly impacts the precision of speech decoding. In essence, the extraction of ultrasonic tongue contours plays a critical role in ensuring effective language communication.

Research indicates that tongue contours serve as an invaluable foundation for the quantitative analysis of speech, with data obtained from these contours facilitating the advancement and comprehension of speech models [3,4]. Ultrasonic tongue contour extraction can dynamically capture the tongue’s position across various phonetic expressions and depict the movements responsible for sound transitions during articulation [5]. This process is integral to nearly all silent speech applications and has become an essential prerequisite. The precision of ultrasonic tongue contour extraction significantly impacts the overall accuracy of speech analysis tasks, while its real-time execution influences the process’s efficiency. Consequently, the identification of a method for ultrasonic tongue contour tracking that is both accurate and rapid is of paramount importance.

Currently, automatic tongue contour tracking presents significant challenges. In the context of ultrasonic tongue imaging, the process is persistently marred by high speckle noise [6]. Obstructions caused by the hyoid and jawbones sometimes impede ultrasound waves, while the tongue’s muscle fibers’ low reflectivity results in incomplete echo paths and sagittal contours [7]. Moreover, images capturing the soft tissue structure of the tongue during positional transformations often contain artifacts, thus rendering contours indiscernible [8]. From the extraction methodology standpoint, the precision of tongue contour delineation is heavily contingent upon the ultrasonic data’s quality and the chosen contour tracking algorithm [5]. Additionally, the extraction process’s speed is hampered by the inherently semiautomatic or manual nature of current methods. Prior research has seldom prioritized speed, and there are no established industry standards for it, with only a maximum speed of 29.8 frames per second (fps) for tongue contour extraction being reported.

A myriad of technologies, including active contour models [9,10,11,12], graph-based techniques [13], and machine learning approaches [14,15], have been employed for tracking tongue contours in ultrasonic imagery. Initially, these methods necessitated manual tagging for initialization, thus rendering real-time tracking by established software like EdgeTrak 1.0.0 [10] infeasible. The advent of deep learning (DL) has garnered significant interest, with convolutional neural networks [16] being recognized for their robust feature extraction capabilities, which are essential for tasks such as ultrasonic tongue contour tracking. Deep confidence networks and autoencoders have also demonstrated promising outcomes [17,18]. DeepLabv3 [19] has shown outstanding performance in the field of semantic segmentation. It effectively captures multiscale contextual information by using atrous convolution and atrous spatial pyramid pooling. This approach provides new methods for segmenting complex scenes, including medical images. Subsequent research highlighted a direct correlation between the efficacy of DL techniques and both the size of the training dataset and the model’s complexity [20,21]. This introduces a delicate balance between training sample volume and network parameter quantity. Achieving high-accuracy extractions mandates ample semantic and detailed information from segmentation networks [22]. Deepening network parameters and enhancing input image resolution for precise segmentation exponentially increases computational demand and diminishes efficiency [23]. Nevertheless, U-Net [24] has proven capable of delivering commendable segmentation outcomes in medical imagery without labeled training data, thus setting a benchmark in the field [25]. Despite this, its deep multilayer architecture significantly taxes computational resources during both training and testing phases. This poses a challenge for real-time ultrasonic tongue contour tracking.

Recent advancements in DL have increasingly emphasized attention mechanisms, which are celebrated for their efficient use of computational resources. The concept, inspired by the human attention system—a pivotal aspect of perception [26,27,28,29]—enables deep convolutional neural networks (DCNNs) to hasten the learning process, discern crucial features more effectively, and bolster model robustness [30]. For instance, Kaul et al. [31] introduced FocusNet, thus integrating attention with a fully convolutional network to segment medical images through feature maps from a distinct convolutional autoencoder. Additionally, incorporating an attention gate (AG) into U-Net’s skip connections has been suggested to refine pancreatic segmentation’s accuracy and sensitivity [32]. Hu et al. [33] explored channel response recalibration within SENet by explicitly modeling channel interdependencies. Similarly, Woo et al. [34] developed the Convolutional Block Attention Module (CBAM), a lightweight, nearly resource-neutral module capable of adaptively refining features from intermediate variables. In ultrasonic tongue contour extraction, the contour line is a minuscule portion of the entire image. Focusing on this target area not only speeds up training but also clarifies object representation and emphasizes detail [35]. Nonetheless, given the potential for fuzzy boundaries and irregular shapes in ultrasonic tongue images, relying solely on a singular attention mechanism might not suffice for effective tongue contour segmentation.

This study introduces a novel feature extraction network model, DAFT-Net, which is enhanced by entropy and information theory principles. It is designed to optimize both accuracy and efficiency, thus addressing the stated objectives, challenges, and technical frameworks. The primary contributions of this work include the following:

- The internal structure of U-Net has been meticulously reconfigured by eliminating one convolutional layer from each encoder and decoder block within the U-Net framework, thus significantly reducing computational demand.
- A novel attention learning module has been developed featuring a synergistic integration of the CBAM and AG modules. This module serves as a comprehensive attention mechanism with dual functionality, thus supplanting the traditional skip connections to enhance the network’s feature representation capabilities.
- The DAFT-Net model’s performance was rigorously evaluated against other neural networks using metrics such as Intersection over Union (IoU), loss, and processing time across three ultrasound datasets: the NS, TJU, and TIMIT datasets. The model achieved 94.93% accuracy and a processing speed of 34.55 ms per image on the NS dataset.

The rest of the manuscript is organized as follows: Section 2 describes the proposed DAFT-Net architecture and its components. Section 3 details the data and experimental details. Section 4 presents the experimental results and discussions. Section 5 validates the ablation experiments. Finally, Section 6 concludes the study and discusses future work.

## 2. Methods

Drawing inspiration from U-Net, Attention U-Net, the Spatial Attention Module, and Gate Attention, this paper introduces DAFT-Net. The network’s architecture is meticulously detailed in the sections that follow.

### 2.1. The Proposed DAFT-Net

**Figure 1:**
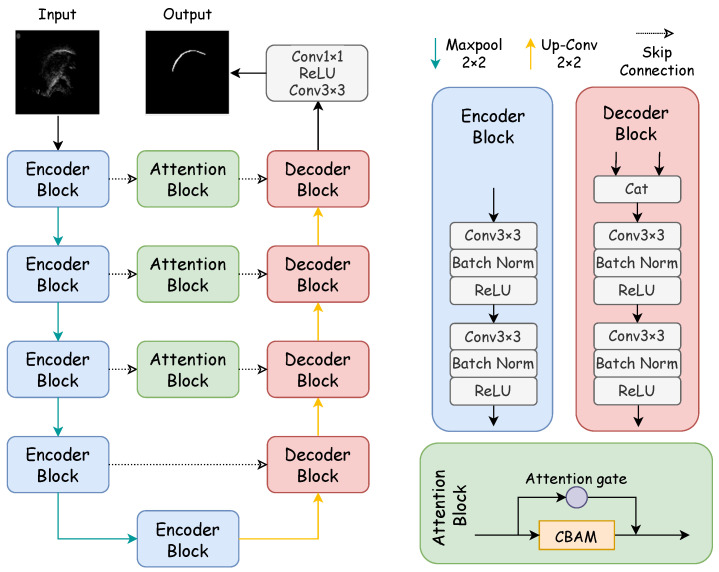
Structure of the DAFT-Net network. The network is mainly composed of a coding path, decoding path, and comprehensive attention modules. The encoder block consists of two convolution blocks, while the decoding block is made up of a concatenation module and two convolution blocks. The concatenation module concatenates the features from the previous layer with the features of the encoder block at the same level. The attention block consists of an attention gate and CBAM.

Figure 1 showcases the architecture of the proposed DAFT-Net, with its internal components to be elaborated in subsequent sections. Our method enhances DAFT-Net by integrating entropy-based optimization techniques, thus focusing on reducing data redundancy and improving feature selection through information-theoretic criteria. In DAFT-Net, we optimize feature selection by minimizing the mutual information of the network outputs. This ensures that the network focuses on the most important features and reduces data redundancy. Specifically, we introduce a mutual information-based regularization term in each attention module. This enables the network to automatically learn to reduce redundancy and highlight key features during the training process.

Specifically, the network’s CBAM and AG modules are fine-tuned to minimize entropy, thus facilitating a more efficient encoding and decoding of ultrasound tongue images. This approach allows for a refined focus on relevant features, thus significantly improving the network’s performance in contour extraction tasks. Mirroring the structure of traditional U–encoder–decoder networks, DAFT-Net comprises an encoding path, a decoding path, and comprehensive attention modules. In Figure 1, rectangles of varying colors denote distinct modules: blue for the encoder module, pink for the decoder module—both forming the network’s symmetric structure—and green for the comprehensive attention module. Each convolution block within these modules consists of a convolutional layer (ConvBlock), a batch normalization layer (BN), and a rectified linear unit (ReLU).

In the encoding path, feature channels double with each downsampling step, thus paralleling the 2 × 2 transpose convolution upsampling in the decoding path, where the number of feature channels is halved. The comprehensive attention module first processes the encoded information. It refines the feature map across different scales while emphasizing noise filtration and elucidating spatial and channel relationships. By using attention mechanisms to filter features, the decoder can learn task-relevant features more effectively, thereby improving segmentation accuracy. The network culminates in a 1 × 1 convolution followed by a sigmoid activation function, thus producing the final segmentation map for the ultrasonic tongue contour.

### 2.2. Network Internal Design

#### 2.2.1. U-Net

The U-Net architecture comprises an encoding path, a decoding path, and skip connections. Within the encoding path, each set of two convolutional layers is followed by a max pooling operation, thus utilizing a 2 × 2 window with a stride of two. Subsequent to every downsampling stage, the feature channels double. Conversely, the decoding path employs a 2 × 2 convolutional layer at each upsampling point, which is activated via a ReLU function. Moreover, each upsampling step integrates the corresponding feature map from the encoding path through skip connections, thus enhancing detail preservation. The network’s final layer features a 1 × 1 convolutional layer, thereby converting the 64-channel feature map into an output corresponding to the classification categories. The overall structure of the U-Net is depicted in Figure 1.

Within the U-Net framework, feature maps retrieved from the encoding path are concatenated with newly generated feature maps during the upsampling phase. This facilitates the fusion of feature images. This process ensures the maximal preservation of feature information through the downsampling stages of the encoding path, thereby enabling precise localization of the segmentation target. To address the challenge of learning from small datasets, data augmentation techniques are applied to expand the dataset before inputting it into the network. Specifically, transformer packages are used to adjust saturation, brightness, and contrast based on random probabilities. Additionally, random rotations and flips are applied to the training images to enhance variability and robustness. Consequently, this study adopts the U-Net architecture as the foundational model for ultrasonic tongue contour extraction.

#### 2.2.2. U-Net Simplified Design

In the U-Net framework, the inclusion of two consecutive convolutional layers aims to expand the receptive field, thereby enhancing feature detection capabilities. However, these additional layers significantly increase the parameter count during the encoding and decoding stages, thus impacting the network’s inference speed. To enhance architectural efficiency without compromising accuracy, this study proposes a modification: the removal of one convolutional layer at each level within the codec convolution block of the original U-Net model. This adjustment maintains accuracy on par with the unaltered model. The architectural modifications are illustrated within the codec block structure depicted in Figure 1. The efficiency and effectiveness of this streamlined approach will be substantiated through ablation studies detailed in Section 5.

### 2.3. Integrated Attention Module

The integrated attention module, marked as the green block in Figure 1, stands as the cornerstone of this network model, thus incorporating both AG and CBAM modules. The fusion of these modules’ outputs enriches the U-Net with parallel encoded feature information, thereby refining the materials available for precise decoding tasks. The AG module reduces noisy and irrelevant responses typically introduced by skip connections. It prioritizes relevant information extracted at a coarse scale. It streamlines the merging of pertinent activations immediately before the concatenation process, thus enabling the selective activation of neurons for both propagation directions. By gleaning additional information from each encoded and decoded path within sub-AGs, it produces a refined output that bypasses the conventional joining process. The AG’s mechanism, akin to a nonlocal block, undergoes a linear transformation devoid of spatial constraints and employs downsampling to diminish the resolution of the feature map. This approach contributes to a reduction in both the parameter count and the computational demands of the network.

Conversely, the CBAM module focuses on dynamically accentuating or attenuating intermediate features to highlight the significance and placement of the target object accurately. Through its dual-attention mechanisms—channel and spatial—it analyzes an input image to yield complementary attention, thus concentrating on feature content and spatial location, respectively. Specifically, the channel attention hones in on the ultrasonic tongue contour, the primary subject of study, whereas the spatial attention zeroes in on pixel positions aligned with the distinct white sagittal tongue contour [35].

**Figure 2:**
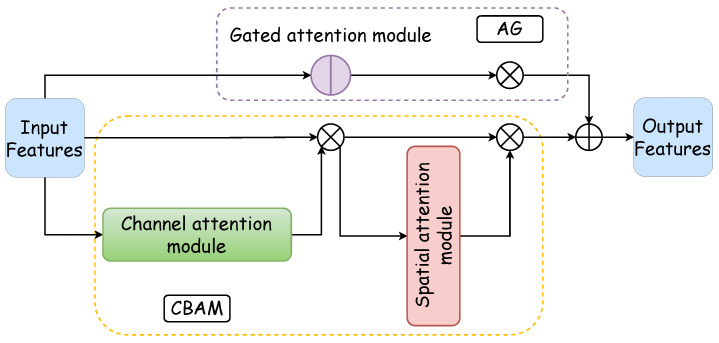
Comprehensive attention module. The comprehensive attention module is composed of a gated attention module and a CBAM in a parallel manner. The CBAM consists of a channel attention module and a spatial attention module, thus forming a dual attention mechanism.

The integrated attention module, which is a parallel configuration of the AG and CBAM, excels in feature processing across distinct dimensions. Moreover, it achieves precise and swift feature localization through the synergistic interaction between the AG and the CBAM. Figure 2 illustrates the architecture of the integrated attention module: the purple box delineates the AG’s internal structure, the yellow dotted box outlines the CBAM’s internal makeup, the blue box represents the CBAM’s channel attention component, and the red box denotes the spatial attention segment of the CBAM. The functionality and design of these two pivotal internal modules of the integrated attention mechanism are expounded upon in the subsequent discussion.

#### 2.3.1. Gated Attention

**Figure 3:**
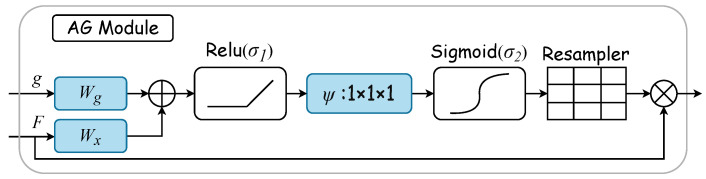
AG module. The gated vector *g* specifies the region of interest. Linear transformations $W_{x}$ and $W_{g}$, along with an offset ψ, embody the AG’s parameter set. These transformations are executed via 1×1×1 convolution across the input tensor’s channels, thus leading to a vector-based connection notation. The AG’s output merges input elements with attention coefficients through elementwise multiplication.

Gated attention mechanisms select specific spatial regions by analyzing contextual information alongside activations from gated signals (g), which are derived from a broader scale. The gating signal (g) is a dynamically computed feature vector used to control the focus of the network’s attention. It determines the importance of each feature in the final feature map by considering contextual information. Figure 3 displays the AG attention module’s internal structure. Here, input features $\left(x^{l}\right)$ are modulated by a concern coefficient (α) related to the resampling grid, with refinement achieved through trilinear interpolation. Trilinear interpolation is an efficient data upsampling technique. This technique interpolates in three-dimensional space to upscale low-resolution attention weights to match the size of high-resolution feature maps. The attention coefficient $\alpha_{i}$, ranging from 0 to 1, pinpoints critical image areas for focused analysis. The “attention coefficient” refers to the values computed by the attention gate mechanism to identify salient image regions and prune feature responses, thus preserving only the activations relevant to the specific task. For each pixel, the gated vector $g_{i}\in R^{F_{g}}$ specifies the region of interest, as detailed in Equations (1) and  (2). The ReLU function is denoted by $\sigma_{1}$, while $\sigma_{2}\left(x_{i,c}\right)=\frac{1}{1+\exp(-x_{i,c})}$ represents the sigmoid activation function. Linear transformations $W_{x}\in R^{F_{l}\times F_{int}}$ and $W_{g}\in R^{F_{g}\times F_{int}}$, along with an offset $\psi \in R^{F_{int}\times 1}$ and $b_{\psi }\in R$, embody the AG’s $\Theta_{\mathrm{att}}$ parameter set. These transformations lead to a vector-based connection notation for cascaded features between $x^{l}$ and *g* within the intermediary space of $R^{F_{int}}$. The AG’s output merges input elements with attention coefficients through elementwise multiplication, as formulaically represented in Equation (3). In this study, the AG calculates a singular scalar focus value for each pixel vector $x_{i}^{l}\in R^{F_{l}}$, with $F_{l}$ indicating the number of feature maps at layer *l*.

(1) $$q_{\mathrm{att}}^{l}=\psi^{T}\left(\sigma_{1}\left(W_{x}^{T}x_{i}^{l}+W_{g}^{T}g_{i}+b_{g}\right)\right)+b_{\psi },$$

(2) $$\alpha_{i}^{l}=\sigma_{2}\left(q_{\mathrm{att}}^{l}\left(x_{i}^{l},g_{i};\Theta_{\mathrm{att}}\right)\right),$$

(3) $${\hat{x}}_{i,c}^{l}=x_{i,c}^{l}\cdot \alpha_{i}^{l}.$$

The AG module dynamically learns to focus on medical image structures of diverse shapes and sizes. It uses an AG-filtered model to emphasize features pertinent to specific tasks through implicit learning and attenuates irrelevant image areas [36]. In ultrasonic tongue contour extraction, the AG plays a crucial role in accentuating essential contours and diminishing nonessential pixels. Thus, integrating the AG into the extraction network is anticipated to substantially enhance network efficiency.

#### 2.3.2. CBAM

**Figure 4:**
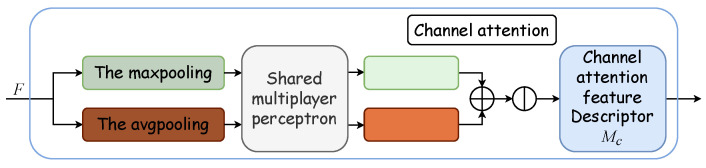
Channel attention module. The channel attention module synthesizes two distinct spatial context descriptors through the aggregation of feature map spatial information via average and maximum pooling, thus yielding the average pooling feature $F_{\mathrm{avg}}^{c}$ and the maximum pooling feature $F_{\max}^{c}$. These descriptors are then processed by a shared multilayer perceptron (MLP) with a hidden layer to produce a channel attention map $M_{C}\in R^{C\times 1\times 1}$.

Outlined within the yellow dotted box in Figure 2, the Convolutional Block Attention Module integrates a channel attention module with a spatial attention module. The channel attention mechanism formulates an attention map by exploring interchannel relationships. Initially, it synthesizes two distinct spatial context descriptors through the aggregation of feature map spatial information via average and maximum pooling, thus yielding the average pooling feature $F_{\mathrm{avg}}^{c}$ and the maximum pooling feature $F_{\max}^{c}$. These descriptors are then processed by a shared network, which is typically a multilayer perceptron (MLP) with a hidden layer, to produce a channel attention map $M_{C}\in R^{C\times 1\times 1}$. To optimize parameter efficiency, the hidden layer’s dimensionality is reduced to $R^{\frac{C}{r}\times 1\times 1}$, with *r* denoting the reduction ratio. Subsequently, the outputs for each descriptor, obtained after passing through the shared network, are aggregated via elementwise summation. The formulation for channel attention is detailed in Equation (4), where σ denotes the sigmoid activation function, and $W_{0}\in R^{\frac{C}{r}\times C}$ and $W_{1}\in R^{C\times \frac{C}{r}}$ represent the MLP’s shared weights, with $W_{0}$ succeeded by the ReLU activation function. Figure 4 displays the schematic of the channel attention module.

(4) $$\begin{array}{cc} M_{C}\left(F\right) & =\sigma (\mathrm{MLP}(\mathrm{AvgPool}(F))+\mathrm{MLP}(\mathrm{MaxPool}(F)))\\ \; & =\sigma \left(W_{1}\left(W_{0}\left(F_{\mathrm{avg}}^{c}\right)\right)+W_{1}\left(W_{0}\left(F_{\max}^{c}\right)\right)\right). \end{array}$$

**Figure 5:**
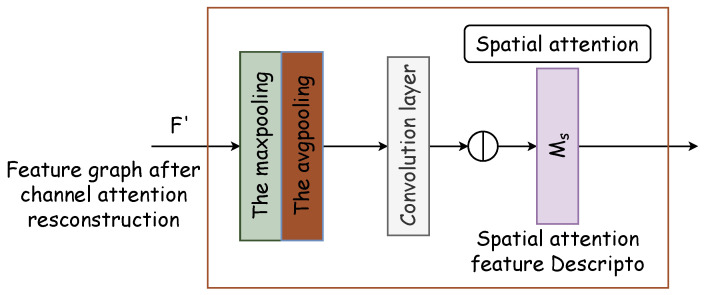
Spatial attention module. Two pooling operations compile channel information of the feature map along the channel axis, thus producing two-dimensional effective feature descriptors $F_{\mathrm{avg}}^{s}$ and $F_{\max}^{s}$. Subsequently, these descriptors are concatenated and processed through a standard convolutional layer to form a 2D spatial attention map $M_{s}\left(F\right)$.

The spatial attention module generates a spatial attention map by leveraging the spatial relationships of features. It distinguishes itself from the channel attention by focusing on the locational information of the target features, thereby complementing channel attention. The spatial attention module’s internal architecture is depicted in Figure 5. Initially, two pooling operations compile the channel information of the feature map along the channel axis, thus producing the two-dimensional effective feature descriptors $F_{\mathrm{avg}}^{s}\in R^{1\times H\times W}$ and $F_{\max}^{s}\in R^{1\times H\times W}$. Subsequently, these descriptors are concatenated and processed through a standard convolutional layer to form a 2D spatial attention map $M_{s}\left(F\right)\in R^{H\times W}$, which designates areas to be highlighted or de-emphasized for feature extraction. The methodology for calculating spatial attention is delineated in Equation (5).

(5) $$M_{s}\left(F\right)=\sigma \left(f_{7\times 7}\left(\left[\mathrm{AvgPool}(F);\mathrm{MaxPool}(F)\right]\right)\right)=\sigma \left(f_{7\times 7}\left(\left[F_{\mathrm{avg}}^{s};F_{\max}^{s}\right]\right)\right).$$

Within the network, the CBAM processes encoded information by addressing both the channel and spatial relationships, thus representing the features to be extracted through a feature vector. Unlike the original U-Net, where full-scale information is directly transferred to the decoding block via skip connections, the modified approach determines the key feature positions first. As a result, with CBAM integration, U-Net selectively channels crucial information to the decoding stage. It enables pixel reconstruction by combining significant feature information at this scale with decoding inputs from other scales to finalize the prediction. The CBAM not only expedites the decoding process but also enhances accuracy by pinpointing essential feature locations, thereby improving the precision of pixel recovery.

## 3. Experiment

### 3.1. Data Preparation

The dataset utilized in this study encompasses the NS dataset, TJU dataset, and TIMIT dataset. The NS dataset comprises 3926 video frames featuring two American English speakers: a male and a female enunciating “The North Wind and The Sun”. The TJU dataset originates from a laboratory at Tianjin University, which was collected from four male native Chinese speakers who recorded real-time ultrasound tongue videos in a professional noise-cancelling environment. The TIMIT dataset represents a comprehensive collection of continuous English speech recorded by native speakers from various regions across the United States, thus encompassing diverse dialects. Each dataset includes ultrasound frames that provide a midsagittal view of the tongue movement during speech, with a resolution of 480 pixels × 640 pixels. These ultrasound frames have been digitized as 2D matrices, where each column corresponds to the ultrasonic reflection intensity along a singular scan line. The ground truth masks have been manually drawn by experienced radiologists to ensure accurate labeling of tongue contours.

**Figure 6:**
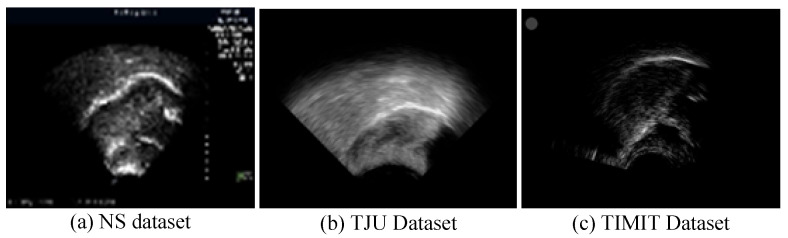
Imaging results of the ultrasonic transducer, with (**a**–**c**) representing samples from the NS dataset, TJU dataset, and TIMIT dataset, respectively. The prominent line at the center represents the tongue’s lateral contour, thus extending from the tongue’s root on the left to its tip on the right.

In ultrasound recordings, the tongue’s contour is delineated by the reflection of ultrasonic waves at the boundary between the tongue and the air above it, thus manifesting as a luminous band. Figure 6 displays ultrasound transducer imaging results, with (a) to (c) representing samples from the NS dataset, TJU dataset, and TIMIT dataset, respectively. The NS dataset features a white sagittal contour that stands out more clearly against the background than those in the other two datasets. In the TJU dataset, the tongue’s contour significantly merges with the surrounding oral cavity, thus making segmentation notably more challenging compared to the other datasets. Meanwhile, the TIMIT dataset’s tongue contour is slender and lacks uniform clarity along its entire length, thus posing difficulties in segmenting the complete tongue line. Although it is less blended with the oral environment, these characteristics still present challenges.

### 3.2. Data Preprocessing

**Figure 7:**
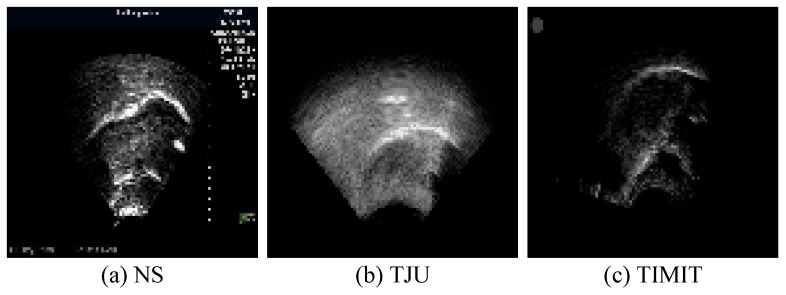
Sample preprocessing results from the tongue datasets following the preprocessing operation.

The white pixels delineating the tongue contour constitute merely 2% of the total pixels in each image. The absence of a clear reference for the tongue’s soft tissue structure results in contours characterized by burrs and discontinuities. This lack of smooth processing can adversely affect the accuracy of ultrasonic tongue contour extraction. To mitigate this, the datasets have been enriched with supplementary information, thereby ensuring that the ground truth encompasses more comprehensive knowledge. This enhancement significantly aids in refining feature extraction accuracy, thereby facilitating subsequent speech research endeavors. Additionally, to augment the datasets’ recognition rate within the network, data augmentation techniques were applied prior to network input. Specifically, transformer packages adjusted saturation, brightness, and contrast based on random probabilities, thus applying random rotations and flips to the training images. These augmentations were executed through the Compose and Oneof functions. Moreover, to enable precise feature extraction from ultrasonic datasets of various sizes and origins, uniform preprocessing was conducted before network training. For this experiment, the images across all datasets were resized to 96 pixels × 96 pixels. Figure 7 illustrates images from the tongue datasets following this preprocessing regimen.

### 3.3. Experimental Implement

The experiment was conducted on a high-performance NVIDIA Tesla V100 GPU training server equipped with 32 GB of video memory, an 8-core CPU, and 40 GB of system memory, thus ensuring a consistent hardware environment for both training and testing phases. The computational setup utilized the Windows 10 operating system, with Python 3.6 serving as the programming language. The design and debugging of the neural network architecture were performed within the PyTorch 1.6.0 framework, thus leveraging its extensive open-source resources.

### 3.4. Evaluation Metrics

In this study, loss, Intersection over Union (IoU), and processing time serve as metrics to evaluate the network’s segmentation efficacy.

Loss and IoU are crucial for assessing the precision of feature extraction in segmentation tasks. Loss, defined as binary crossentropy, quantifies the discrepancy between predicted outputs and actual targets. It is a fundamental metric in target detection, thus providing insight into the model’s performance. IoU, on the other hand, gauges the pixel-level congruence between the segmented tongue contour and the true mask, thus serving as a direct measure of segmentation accuracy. The loss calculation is described by Equation (6), where *N* denotes the total pixel count, $y_{i}$ denotes the true label, ${\hat{y}}_{i}$ denotes the predicted label, and L(·) denotes the mapping function. The IoU’s formulation, shown in Equation (7), involves $y^{*}$ as the prediction, $\tilde{y}$ as the ground truth, *c* as each pixel, and $J_{c}\left(\cdot \right)$ as the mapping function. A smaller loss value and a larger IoU indicate enhanced segmentation precision.

(6) $$L_{BCE}=L\left(y_{i},\overset{\wedge }{y_{i}}\right)=-\frac{1}{N}\sum_{i=1}^{N}\left[y_{i}\log\overset{\wedge }{y_{i}}+\left(1-y_{i}\right)\log\left(1-\overset{\wedge }{y_{i}}\right)\right],$$

(7) $$J_{c}\left(y^{*},\tilde{y}\right)=\frac{\left|\left\{y^{*}=c\right\}\cap \left\{\tilde{y}=c\right\}\right|}{\left|\left\{y^{*}=c\right\}\cup \left\{\tilde{y}=c\right\}\right|}.$$

In this study, the processing time was utilized to assess the network’s real-time performance capabilities. The experimental datasets consist entirely of image sets, analogous to frames in video processing, thus rendering the input to the network as static images, with the testing datasets also comprising image sets. Consequently, the time required to test a single image was chosen as a proxy for the network’s testing speed. This metric, denoted as Time, is measured in milliseconds and corresponds to the duration needed to process each image frame. Time not only reflects the speed at which segmentation tasks are completed but also serves as a critical metric for evaluating the network’s performance. Essentially, the shorter the processing time, the faster the image is processed, thus indicating superior real-time performance capabilities in practical applications.

### 3.5. Experiment Setting

The dataset distribution for the study was as follows: 50% for training, 20% for validation, and 30% for testing. The experiment utilized a batch_size of 32, a learning rate of 0.001, 100 iterations, a momentum of 0.9, and employed the Adam optimizer. An early stopping mechanism was incorporated to halt the training process using the validation set for monitoring. This ensured that the model achieving the lowest average loss on the validation set would be selected as the optimal model for further evaluation.

Given U-Net’s prevalence as a cornerstone medical segmentation network and a foundational model for numerous image recognition architectures, this study devised several comparative experiments centered around U-Net. It assessed the efficacy of Unet++, SA-UNet, and SegAN across the three datasets by examining loss, IoU, and time metrics to compare their accuracy and real-time feature extraction performance. Furthermore, to ascertain the contributions of the DAFT-Net network proposed in this research towards network simplification, the AG and CBAM modules were integrated to enhance U-Net’s functionality. This was followed by an ablation study to evaluate their impact.

## 4. Experimental Results and Discussion

**Figure 8:**
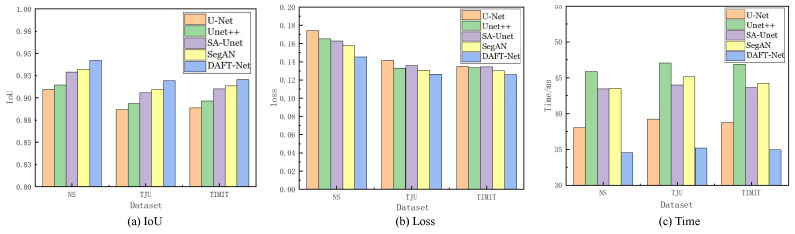
Comparison of IoU, loss, and Time metrics for U-Net, UNet++, SA-UNet, SegAN, and DAFT-Net, respectively, across the NS, TIMIT, TJU datasets.

**Figure 9:**
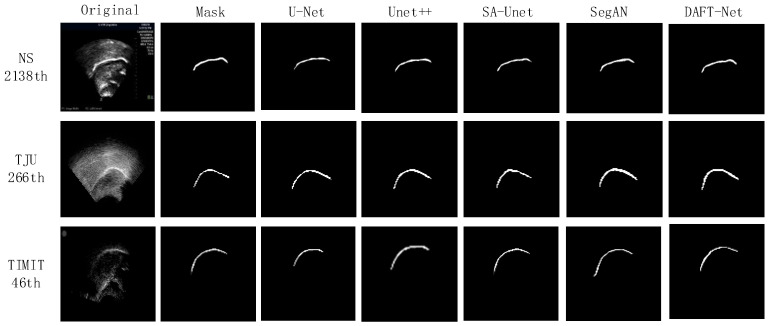
Visual comparison results between our DAFT-Net and other four compared methods on the NS, TIMIT, TJU datasets.

Using the U-Net network as a benchmark, UNet++, SA-UNet, and SegAN were included in comparative experiments. The comparative results are detailed in Table 1, where the IoU, loss, and Time metrics for each network across different datasets are enumerated. The $Q_{\mathrm{IoU}}$ and $Q_{\mathrm{Loss}}$ represent the ranking values according to quartiles for the methods with the highest IoU and the lowest loss values, as is similar to [37]. Figure 8 graphically illustrates these metrics for five networks across the three datasets via bar charts: (a) for IoU, (b) for loss, and (c) for Time. Figure 9 showcases the segmentation effects of the five networks arranged in a matrix of three rows and seven columns. A frame from each dataset was randomly selected for evaluation: frame 2138 from the NS dataset, frame 266 from the TJU dataset, and frame 46 from the TIMIT dataset. The first column presents the original sample images, the second column shows the corresponding mask images, and the third to seventh columns display the predictive outcomes from U-Net, UNet++, SA-UNet, SegAN, and DAFT-Net, respectively. This study conducted a comprehensive analysis of each network using these three metrics and segmentation effect diagrams, thus aiming to identify a network that balances high accuracy with rapid processing. Although the visual results of various methods appear similar, DAFT-Net had an advantage in enhancing edge definition. This is crucial for subsequent medical applications such as real-time speech analysis and speech disorder assessment.

Regarding the dataset performance, the five network groups ranked as follows: NS dataset > TIMIT dataset > TJU dataset. This ranking underscores the significant influence of dataset feature clarity on network extraction effectiveness. It also validates the anticipated challenges in feature extraction across the three datasets, as was predicted in Section 3.1.

The network comparison reveals that UNet++ enhanced U-Net by extending the selection of feature scales and enriching semantic information through a nested network structure. However, its fragmented and complex internal structure significantly increased the operational parameter count, thus presenting challenges for real-time ultrasonic tongue imaging applications. Notably, UNet++ demonstrated a 7.85 ms (20.64%) longer processing time on the NS dataset compared to U-Net, thus highlighting its limitations in real-time performance.

SA-UNet, on the other hand, reimagines U-Net by incorporating a spatial attention mechanism [36], thus showcasing how attention mechanisms can refine U-Net’s architecture. Despite its modest time improvement over U-Net, SA-UNet exceled in accuracy, thus underscoring the benefits of spatial attention in segmentation tasks.

SegAN, a generative adversarial network with segmentation capabilities, achieved stable feature extraction with minimal training samples. Nevertheless, the adversarial process, involving the generation of false samples to deceive the discriminator, prolonged the extraction timeline [38]. Compared to its counterparts, SegAN underperformed in the processing speed relative to U-Net, matched SA-UNet, and surpassed only UNet++. DeepLabv3 is based on atrous convolution and spatial pyramid pooling. As shown in Table 1, compared to non-UNet methods such as SegAN and DeepLabv3, our improved UNet-based method demonstrated greater advantages.

The approach introduced in this study involves integrating an attention gate module into the SA-UNet framework and substituting its original spatial attention mechanism with a CBAM that addresses both spatial and channel relations. While this adjustment ostensibly complicates the network’s architecture, it facilitates swift feature extraction—a capability substantiated by the experimental outcomes. The AG and CBAM modules primarily expedite the identification of crucial features, thereby enhancing segmentation and extraction processes. Despite their intricate functionality, the computational demand and resource consumption of these modules are minimal, thus owing to the efficiency of the internal connection and convolution operations. Consequently, this dual-module integration significantly refines the accuracy of ultrasonic tongue contour extraction without extending the network’s operational duration. Moreover, this streamlined network, augmented by the synergistic function of both attention modules, achieved notable segmentation speeds—94.23% accuracy within 34.55 ms/frame on the NS dataset, 91.95% accuracy within 35.17 ms/frame on the TJU dataset, and 92.06% accuracy within 34.93 ms/frame on the TIMIT dataset—thus demonstrating its efficacy in rapid segmentation tasks.

## 5. Ablation Experiment Validation

This paper streamlined the U-Net architecture by reducing its layers and incorporated both the AG and CBAM modules via a parallel configuration, thus resulting in the creation of DAFT-Net. The design process focused on three pivotal modifications: (1) layer reduction in U-Net; (2) integration of the AG module; and (3) integration of the CBAM module. To evaluate the individual impact of these components on the baseline U-Net network, dedicated ablation studies were conducted.

**Figure 10:**
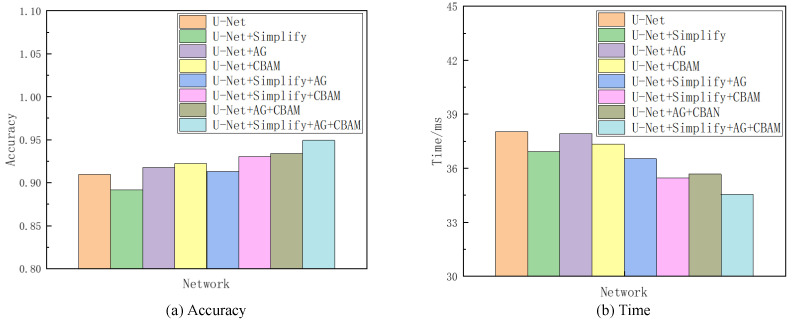
Comparison of accuracy and Time metirics of the ablation experiments on the NS dataset.

Given the superior segmentation performance on the NS dataset in terms of both accuracy and speed, as detailed in Section 4, it was selected as the reference for the subsequent ablation analysis. This investigation encompassed seven sets of comparative experiments. The network variant derived from U-Net by excising one convolutional layer at each codec level is denoted as “Simplify” in the analysis. Combining “Simplify”, AG, and CBAM with U-Net through all possible permutations yielded seven distinct networks, thus excluding the original U-Net. The evaluative focus of these models’ training and testing phases centered on discerning the contributory value of each element. This value was inferred from comparative analysis, with enhancements in accuracy and processing time being indicative of each component’s efficacy in augmenting the baseline network. Given the project’s emphasis on sagittal tongue contour segmentation, the IoU served as the primary metric for accuracy, while the Time metric quantified the duration each model took to process an identical ultrasonic image. Table 2 compiles the findings from this ablation study, and Figure 10 provides a visual comparative analysis through bar charts, thus showcasing each model’s performance on the NS dataset concerning accuracy and time. Additionally, visualization tests from various networks on frame 2138 of the NS dataset within this ablation framework are itemized in Table 3.

Referencing Table 2 and Table 3, as well as Figure 10, the Simplify version of U-Net exhibited a modest decline in accuracy by 1.8% compared to the original network. However, it showed a processing time improvement of 1.11 ms attributed to reduced convolutional layer parameters and computational load. This Simplify model strikes a good balance between accuracy and speed, thus demonstrating an overall superior performance in ultrasonic tongue contour extraction tasks relative to the original U-Net architecture. The integration of attention modules further enhances both accuracy and efficiency. Specifically, employing the AG module alone outperformed both Simplify and U-Net in terms of accuracy, with its processing time falling between the two. Utilizing the CBAM module independently yielded even better results in accuracy and time than using the AG module alone. Combining both attention modules surpassed the performance achieved with either module individually, with an accuracy increase of 1.6% and a 2.25 ms reduction in processing time compared to the AG module alone. Compared to the CBAM module alone, the dual-module approach improved the accuracy by 1.1% and reduced the processing time by 1.66 ms. The incorporation of Simplify with both modules markedly enhanced the metrics, thus showing a 5.87% improvement in accuracy and a 2.37 ms decrease in processing time compared to using Simplify alone.

The outcomes of the ablation studies underscore the practical viability of balancing accuracy and speed. U-Net, a well-established image segmentation network, exemplifies how a thoughtfully designed lightweight framework can ensure precise feature extraction. This paper advances the network’s architecture by streamlining the original structure and integrating a triple attention mechanism. The introduction of the Simplify, AG, and CBAM modules enhances U-Net’s already formidable capability in ultrasonic tongue contour extraction, thus highlighting the significant benefits of these strategic modifications.

The effectiveness of incorporating entropy and information theory into the DAFT-Net is evident in its improved performance. Our analysis highlights the network’s enhanced capability in reducing entropy and efficiently managing information, thus leading to more precise and faster tongue contour extraction. This advancement not only aligns with the theoretical expectations of entropy and information theory but also enhances the way these concepts are applied in medical image analysis.

## 6. Conclusions

This paper proposes a U-Net-based algorithm for ultrasonic tongue contour image segmentation, thus integrating Attention Gate and Convolutional Block Attention Modules empowered by entropy and information theory. Experimental findings across three datasets demonstrate that incorporating attention mechanisms enhances efficiency. This was achieved either by enhancing the original U-Net’s connectivity or by simplifying the network through reduced convolutional layers. These methods align with the objectives of entropy minimization and information maximization. The combined implementation of these modifications utilizes entropy-based and information-theoretic approaches to accelerate feature extraction and improves segmentation accuracy while conserving computational resources. If successfully applied to actual ultrasonic tongue contour extraction tasks, this method could significantly streamline collaboration with silent speech and other medical fields. It demonstrates the practical applicability of entropy and information theory in enhancing medical image processing. This breakthrough aligns with our research group’s objectives and marks a significant step forward in the interdisciplinary application of information theory and entropy to medical image analysis. 

## Figures and Tables

**Table 1:** Comparative experimental results of IoU, loss, and Time metrics between our DAFT-Net and other four compared methods on the NS, TIMIT, TJU datasets.

Method	Dataset	IoU	Loss	Time (ms)	$Q_{\mathrm{IoU}}$	$Q_{\mathrm{Loss}}$
	NS	0.9098 ± 0.0943	0.1740 ± 0.0387	38.03 ± 6.54	5	6
U-Net	TJU	0.8869 ± 0.0728	0.1415 ± 0.0305	39.25 ± 5.12	5	6
	TIMIT	0.8887 ± 0.0869	0.1346 ± 0.0642	38.76 ± 3.55	5	6
	NS	0.9145 ± 0.0676	0.1653 ± 0.0432	45.88 ± 7.42	4	4
Unet++	TJU	0.8935 ± 0.0784	0.1329 ± 0.0553	47.05 ± 4.25	4	3
	TIMIT	0.8967 ± 0.0535	0.1332 ± 0.0798	46.86 ± 3.54	4	4
	NS	0.9290 ± 0.0627	0.1629 ± 0.0474	43.44 ± 8.38	3	3
SA-Unet	TJU	0.9057 ± 0.0898	0.1356 ± 0.0582	43.97 ± 4.65	3	5
	TIMIT	0.9102 ± 0.0369	0.1344 ± 0.0690	43.65 ± 5.47	3	5
	NS	0.9321 ± 0.0886	0.1577 ± 0.0354	43.52 ± 7.36	2	2
SegAN	TJU	0.9095 ± 0.0613	0.1305 ± 0.0576	45.16 ± 5.82	2	2
	TIMIT	0.9133 ± 0.0445	0.1299 ± 0.0787	44.22 ± 6.41	2	2
	NS	0.9091 ± 0.0629	0.1667 ± 0.0673	44.63 ± 8.91	6	5
Deeplab V3	TJU	0.8853 ± 0.0776	0.1338 ± 0.0885	46.35 ± 6.85	6	4
	TIMIT	0.8876 ± 0.0334	0.1326 ± 0.0694	45.78 ± 5.57	6	3
	NS	0.9493 ± 0.0587	0.1452 ± 0.0565	34.55 ± 4.83	1	1
DAFT-Net	TJU	0.9195 ± 0.0369	0.1262 ± 0.0682	35.17 ± 5.25	1	1
	TIMIT	0.9206 ± 0.0592	0.1258 ± 0.0478	34.93 ± 3.71	1	1

**Table 2:** Ablation study of proposed modules on the NS dataset. Simplify denotes the network variant derived from U-Net by excising one convolutional layer at each encoder level. AG represents the Attention Gate module. The CBAM denotes the Convolutional Block Attention Module. The accuracy means the IoU metrics. The Time metric quantifies the duration each model takes to process an identical ultrasonic image.

Method	Simplify	AG	CBAM	Accuracy	Time (ms)
	×	×	×	0.9098 ± 0.0943	38.03 ± 6.54
	✔	×	×	0.8916 ± 0.0816	36.92 ± 5.81
	×	✔	×	0.9175 ± 0.0728	37.93 ± 8.26
U-Net	×	×	✔	0.9226 ± 0.0682	37.34 ± 6.35
	✔	✔	×	0.9132 ± 0.0553	36.53 ± 7.81
	✔	×	✔	0.9305 ± 0.0636	35.47 ± 6.53
	×	✔	✔	0.9337 ± 0.0534	35.68 ± 5.38
DAFT-Net	✔	✔	✔	0.9493 ± 0.0587	34.55 ± 4.83

**Table 3:** Visualization test results of each network in the ablation experiments (with the 2138th frame of the NS dataset taken as an example). Simplify denotes the network variant derived from U-Net by excising one convolutional layer at each codec level. AG represents the Attention Gate module. The CBAM denotes the Convolutional Block Attention Module.

Model	Result	Model	Result
U-Net	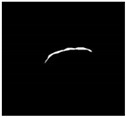	U-Net + Simplify + AG	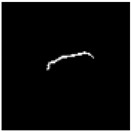
U-Net + Simplify	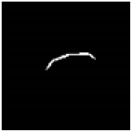	U-Net + Simplify + CBAM	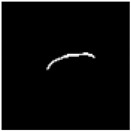
U-Net + AG	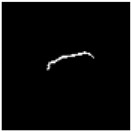	U-Net + AG + CBAM	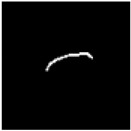
U-Net + CBAM	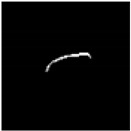	U-Net + Simplify + AG + CBAM	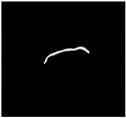

## Data Availability

The NS and TIMIT datasets are datasets are publicly available on the network. The TJU dataset is provided by Tianjin University Laboratory and is not public at present.
